# Modelling biofilm-induced formation damage and biocide treatment in subsurface geosystems

**DOI:** 10.1111/1751-7915.12002

**Published:** 2012-11-20

**Authors:** C C Ezeuko, A Sen, I D Gates

**Affiliations:** Department of Chemical and Petroleum Engineering, Schulich School of Engineering, University of CalgaryCalgary, Alberta, Canada

## Abstract

Biofilm growth in subsurface porous media, and its treatment with biocides (antimicrobial agents), involves a complex interaction of biogeochemical processes which provide non-trivial mathematical modelling challenges. Although there are literature reports of mathematical models to evaluate biofilm tolerance to biocides, none of these models have investigated biocide treatment of biofilms growing in interconnected porous media with flow. In this paper, we present a numerical investigation using a pore network model of biofilm growth, formation damage and biocide treatment. The model includes three phases (aqueous, adsorbed biofilm, and solid matrix), a single growth-limiting nutrient and a single biocide dissolved in the water. Biofilm is assumed to contain a single species of microbe, in which each cell can be a viable persister, a viable non-persister, or non-viable (dead). Persisters describe small subpopulation of cells which are tolerant to biocide treatment. Biofilm tolerance to biocide treatment is regulated by persister cells and includes ‘innate’ and ‘biocide-induced’ factors. Simulations demonstrate that biofilm tolerance to biocides can increase with biofilm maturity, and that biocide treatment alone does not reverse biofilm-induced formation damage. Also, a successful application of biological permeability conformance treatment involving geologic layers with flow communication is more complicated than simply engineering the attachment of biofilm-forming cells at desired sites.

## Introduction

Formation damage, as defined by the Society of Petroleum Engineers, is the reduction in reservoir permeability resulting from infiltration of drilling or treating fluids into the area adjacent to the wellbore (SPE, 2011). Causes of formation damage include fines migration, scaling, gas blockage associated with production, mineral precipitation and microbial biomass generation (Dake, 2001). Formation damage can occur at any time during the life of a production or injection well including workover, stimulation (e.g. conformance treatment; hydraulic fracturing, matrix acidizing), completion, or kill operations (Bennion, 2002). Typically, stimulation operations aim to improve productivity from a damaged formation or a naturally low permeability reservoir. In the context of conformance control, the absolute permeability of the formation may be reduced by infiltration of treating fluid, yet the productivity can be improved. Therefore, to index the impact of formation damage, we measure the reduction in reservoir ‘*productivity*’ rather than ‘*permeability*’. Many causes of formation damage have been investigated. Despite extensive research and general consensus on the challenges of bioclogging in engineered processes, literature dealing with implications of biofilm-induced formation damage in petroleum geosystems is sparse.

### Hydraulic fracturing

The continuous decline in conventional petroleum reserves has motivated development of unconventional hydrocarbon sources (e.g. shale gas, coal bed methane, oil shale) in recent years (Holditch, 2009). In order to increase productivity of these resources, hydraulic fracturing is commonly employed to create and sustain artificial fractures in the rock matrix. Successful hydraulic fracturing operation enlarges the volume of reservoir linked by relatively high permeability medium to the production wellbore. Usually, a multistage fracturing approach is used where enormous volumes of water (> 350 m^3^) are injected at each stage. The large volumes of water are often obtained from surface sources, such as ponds and rivers, which typically always contain significant populations of microorganisms. When injected along with fracture fluid, these microorganisms proliferate in the pore space leading to different forms of biofouling including; bioclogging, microbially induced corrosion, or H_2_S production if the organisms are sulfate reducing bacteria. To prevent excessive microbial growth, biocides (e.g. glutaraldehyde, sodium hypochlorite, 2-bromo-2-nitropropane-1,3-diol) are added to hydraulic fracture fluids to kill a broad spectrum of bacteria.

An ideal biocide is one that is (i) safe to handle, (ii) cost effective, (iii) does not adversely affect fracture fluid characteristics, (iv) does not adversely affect formation fluids, (v) exhibits high antimicrobial activity at low concentrations over the long term and (vi) has minimum environmental impact. Whereas current biocides appear to be relatively effective at controlling microbial growth in the region adjacent to the wellbore over the short term, they may not be ideal at preventing long-term microbial activity. One reason is that bacterial populations are heterogeneous and tend to have a small subpopulation known as ‘persisters’ which are tolerant to biocide treatment (i.e. they can survive exposure to a biocide, but do not grow or proliferate in its presence). However, when not exposed to biocide for a period of time, a proportion of these persister cells revert to non-persister cells capable of proliferation and generation of biofilm. Major implications of biofilm generation are reduced formation productivity and a possible increase in tolerance to biocide treatment (see Anderson and O'Toole, 2008 for a detailed review of biofilm mechanisms of tolerance to biocide). The capacity of biofilms to effectively retain persister cells may contribute to the increased biocide tolerance exhibited by biofilms (Keren *et al*., 2004a). Unfortunately, biocide efficacy is typically tested in culture against planktonic bacteria, thereby making it difficult to predict the efficacy of biocide treatment when biofilm growth occurs in porous media. Ineffective biofilm treatment may, as a result, increase the number of hydraulic fracturing cycles needed during the operational lifespan of a well. This substantially increases the total volume of water used for hydraulic fracturing, and significantly increases the risk of contaminating potable aquifers. Moreover, proper disposal of enormous quantities of produced toxic water is a huge challenge. Therefore, understanding pore scale development of biofilms and actions of biocide during flow through hydraulically fractured porous media is essential to develop more effective biocides and improve biocide delivery strategies that have better long-term efficacy.

### Waterflood permeability conformance

Biofilm growth can also impact waterflood performance (Shaw *et al*., 1985). The promising performance of waterflooding, both for low viscosity/light oil (Hermansen *et al*., 2000), and high viscosity/heavy oil (Beliveau, 2009; Mai and Kantzas, 2010), suggest that more oil field developments will adopt waterflooding. One problem associated with waterflood operations is preferential flow of water through high permeability layers in comparison to low permeability layers (Craig, 1993; Dake, 2001). This means that high permeability zones become more water-rich as flooding continues, and oil in the lower permeability rock is bypassed resulting in reduced productivity. Current conformance control techniques use water-soluble polymer (e.g. polyacrylamide) placement within the reservoir (Fletcher *et al*., 1992; Sorbie and Seright, 1992; Sydansk and Southwell, 1998). However, injection of these water-borne polymers into the reservoir can also lead to significant formation damage, and possibly, loss of production (Sparlin, 1977). In contrast to hydraulic fracturing where any form of biofilm growth is considered detrimental, targeted biofilm growth in water-rich high permeability zones can be used to improve overall waterflooding performance. This is not a new technology – the concept has been explored to improve sweep efficiency (Gullapalli *et al*., 2000; Brown *et al*., 2002). Biofilm growth rates are greater in high permeability zones due to larger flow and advective mass transport of dissolved nutrients, eventually resulting in bioclogging of high permeability zones and, diversion of water into low permeability zones (Raiders *et al*., 1986; 1989).

### Biofilm tolerance to biocides

An increase in tolerance to biocides indicates an increase in the complexity and cost of treating biofilm-related forms of biofouling. One popular theory explaining this tolerance is that the biocide concentration that reaches non-persister cells within biofilms is below a critical biocidal threshold, due either to the reduced diffusive transport of biocides across EPS (extracellular polymeric substances), or the adsorption of positively charged biocides by anionic EPS matrix components (Anderson and O'Toole, 2008). Inhibition of biocide diffusion by EPS matrix accounts for biofilm tolerance to biocide in some cases (Suci *et al*., 1994), but not others (Dunne *et al*., 1993). Even when a biocide eventually reaches a biofilm at a concentration sufficient to kill planktonic bacteria, it may not affect the cells within the biofilm. The micro-environment within the biofilm is altered compared with the planktonic cell environment, and as a result, biofilm cells may have switched to protection mode, with reduced metabolic activities, making them more tolerant to biocides (Anderl *et al*., 2003). Questions regarding the possibility of obtaining misleading results of biocide penetration due to the movement of biocides through gaps in interstices between microcolonies have also been raised (Davies, 2003). These apparently contrasting views suggest that tolerance depends on several factors such as specific bacterial consortia, and transport capabilities of the specific biocide (Brooun *et al*., 2000).

The presence of persister cells has also been proposed as a tolerance mechanism (Spoering and Lewis, 2001; Keren *et al*., 2004b; Lewis, 2005). Persister cells describe a bacterial phenotype that is highly tolerant to antimicrobial treatment. This concept of persister cells has been implemented in some mathematical models (Cogan *et al*., 2005; Roberts and Stewart, 2005; Cogan, 2006), whereby biofilm tolerance to biocide evolves through the production of a subpopulation of tolerant (persister) cells whose gene expression is regulated by the non-persister cell population. In addition, it has recently been suggested that gene expression in persister cells may be regulated by the biocide concentration (Lewis, 2005).

In this paper, we present a mathematical model which, for the first time, incorporates both innate tolerance (i.e. tolerance that is an integral part of biofilm structure and physiology) and biocide-induced tolerance (i.e. factors resulting from biocide exposure) of biofilms during biocide treatment in interconnected porous media under flow conditions. Here, we adopt a pore network modelling approach which implements important pore-level biophysical processes relevant to biofilm evolution in porous media, in a discretized form, by using pores with accurately defined geometrical properties. Also, we explore biofilm evolution during nutrient injection into a high permeability layer that is in communication with an adjacent low permeability layer. This provides insight into how biofilm growth patterns may influence flow diversion. A further objective of the work is to assess a time-lapsed productivity index as an easy to calculate parameter to evaluate biofilm-induced formation damage during permeability conformance treatment. Although the model is generic in nature, presented simulations and discussion are used to explore formation damage applicable to hydraulic fracturing and permeability conformance.

## Model development

The following general assumptions have been made during model development:

- The porous matrix is fixed and incompressible and each pore element can host multiple phases. Nevertheless, the presented simulations consider only a single flowing (aqueous) phase. Biofilm is of constant density and attaches as a homogeneous layer to the surface of the pore wall – therefore, each volume fraction in the presented model equations have the same density.
- Biofilm is monomicrobial, and each cell can exist in a non-persister, dead, or persister state.
- Planktonic cell nutrient consumption and growth is negligible and the Monod (1949) equation can reasonably predict the growth of biofilm bacteria. Also, liquid flow through biofilm is possible.
- A single growth-limiting nutrient is available and both perfectly soluble and non-reactive in water. Within a phase, the diffusion coefficient is homogeneous and isotropic (i.e. *D* = *D*_*x*_ = *D*_*y*_ = *D*_*z*_). Solute diffusion into the solid matrix is negligible. In mathematical terms, spatial resolution within a biofilm is zero-dimensional in this model (i.e. cell components are homogeneous). In addition, concentration gradient of substrate and biocide are zero within a biofilm.
- Nutrient transport can be approximated by advection–diffusion–reaction formulation involving a viscous and incompressible fluid undergoing steady-state laminar flow.
- Transversal transport/mixing processes (in the pore water and the biofilm) are considered to be sufficiently fast and are thus neglected. As a result, a single concentration is defined within a pore.
- Once biofilm attaches to the pore wall, biofilm detachment occurs only when the local shear stress exceeds a predefined critical shear stress. The value of critical shear stress is dependent upon both biofilm physiology and local environmental conditions.
- No flow occurs at boundaries transverse to the principal flow direction (both within pores and at the network level). Also, fixed pressure boundaries are implemented at the network inlet and outlet.

**Fig. 1:**
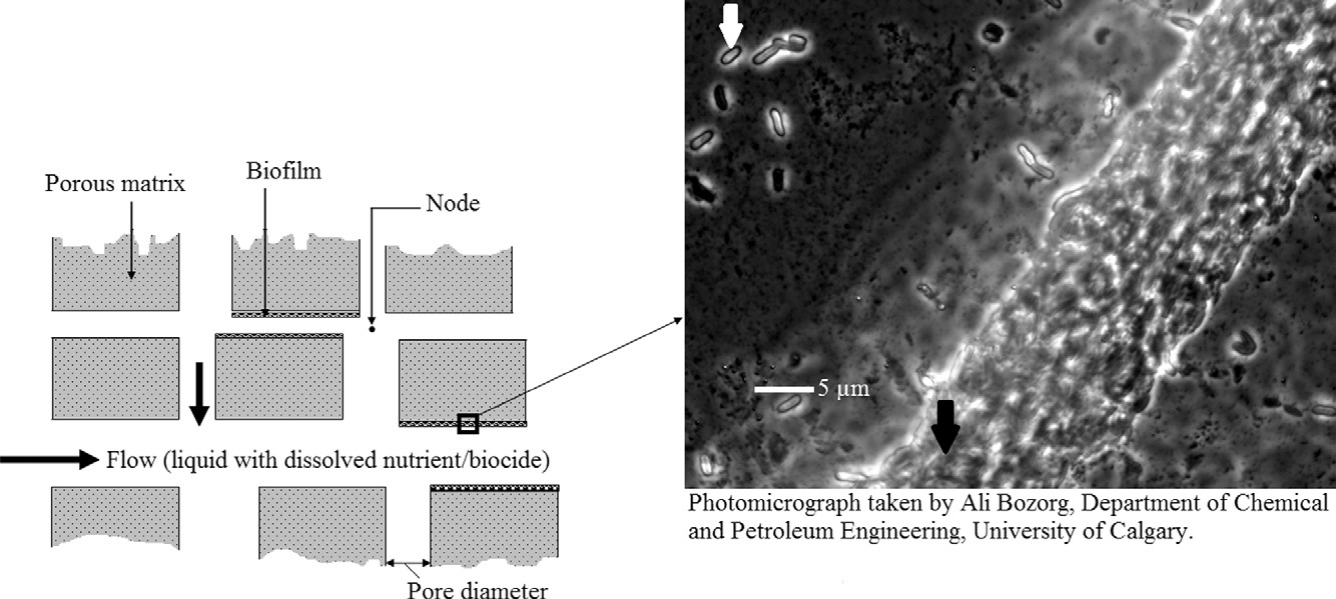
Schematic showing definitions relevant to our pore network model including flow through pore spaces and biofilm adsorption on pore walls. An enlarged picture of a *Pseudomonas fluorescens* biofilm is presented and highlights key components of biofilm in our model, which are cells and EPS matrix. White arrow indicates bacterial cell and black arrow indicates EPS produced by bacteria.

**Fig. 2:**
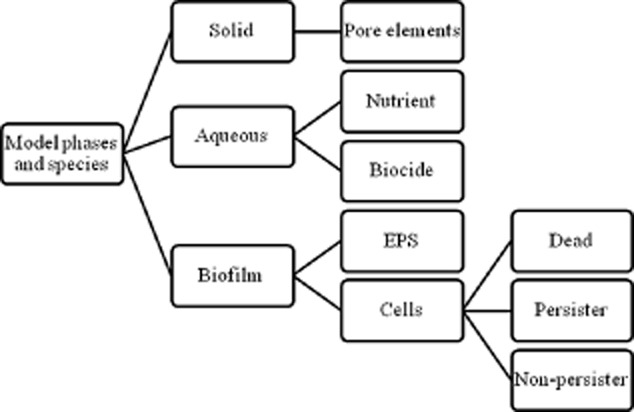
Schematic description of the different phases and species considered at the pore level in our model. Biocide and nutrient are dissolved in the aqueous phase and can be injected simultaneously. EPS stands for extracellular polymeric substance produced by bacterial cells.

We recognize that various other factors (e.g. predation, etc.) can contribute to biomass removal, depending on the system under consideration. Endogenous decay as implemented in our model serves to capture cell death not related to biocide. For modelling purposes, we can either retain these dead cells in the biofilm or consider them now in the bulk phase and eventually removed from the system. This way, biomass can slightly decline over a significantly long period. However, even for laboratory scale bioclogging, it is expected that a bioclogged system remains clogged over fairly long timescales (e.g. Kim and Fogler, 2000) without recourse to different forms of external forces (e.g. shear forces, mechanical forces) or use of a highly reactive biocide. Therefore, retaining cells which died in a biofilm via an endogenous decay mechanism should be well within the limit of reasonable assumptions. Key design limitations of presented model are associated with the assumption of mono-species/mono-nutrient conditions and the assumption of zero-dimensional spatial resolution within a biofilm. In reality, biofilms are usually multispecies colonies and the resultant competition for nutrients can alter bacterial rate of growth. Assumptions of zero-dimensionality within a biofilm implies that the presented model needs further improvement to capture complex spatial distribution of components (e.g. cells, nutrients) within a biofilm and overcome the limitation of planar biofilm and substratum geometry. Figure 1 shows a schematic that describes interactions between flow through interconnected pores and biofilm growth as implemented in our model. The porous medium is treated as a two-dimensional (2D) network of interconnected pore elements (bonds). Each intersection of pore elements is referred to as a node. A constant pressure gradient is applied across the model domain. A summary of pore-level phases and species accounted for in our model is depicted in Fig. 2. Multiple flowing liquid phases can be modelled by adopting the capillary pressure concept (Ezeuko *et al*., 2010). We track temporal evolution of nutrient within each pore element by coupling flow, mass transport (advective and diffusive), and reaction processes.

### Flow model

It has been observed that liquid can flow through biofilms (Stoodley *et al*., 1994), and the effective pseudoviscosity of water flowing through a biofilm can be defined by (Dupin *et al*., 2001):


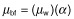
(1)

where *μ_w_* is the viscosity of water flowing in the bulk and *α* is the ratio of the viscosity of water flowing through biofilm to the viscosity of water flowing in the bulk. We assume that a parallel or near-parallel laminar flow of viscous incompressible fluid in each cylindrical pore is governed by a modified form of the Poiseuille equation (Thullner and Baveye, 2008):


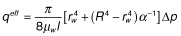
(2)

where *q^eff^* is the effective pore-level volumetric flow rate, Δ*p* is the pressure drop between two nodes connecting a pore element, *l* is the length of the pore element, *R* is the pore radius (zero biofilm thickness), and *r_w_* is the radius of the pore section occupied by bulk water. A mass balance at each node yields:


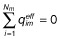
(3)

where *m* is the label of an arbitrary node and *N_m_* is the total number of pore elements connected to that node (for example, *N_m_* = 4 for a fully connected 2D simple rectangular network). By substituting Eq. 2 into Eq. 3 for all nodes, a sparse system of linear equations results with the pressure at each node as the unknowns (Suchomel *et al*., 1998). Pore element flow rates are then calculated from Eq. 2.

### Transport model

We model nutrient and biocide transport within each pore element by using a discretized form of the one-dimensional convection–diffusion–reaction equation:


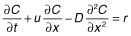
(4)

where *C* is the local nutrient concentration, *D* is the diffusion coefficient, *u* is the mean velocity through the pore element, *x* is the coordinate parallel to the trajectory of the pore element, and *r* is the reaction term associated with nutrient consumption. Also, we treat nutrients in bulk water within a pore element as accessible to the biofilm cells within that pore and as a result, define a single nutrient concentration within a pore element.

**Fig. 3:**
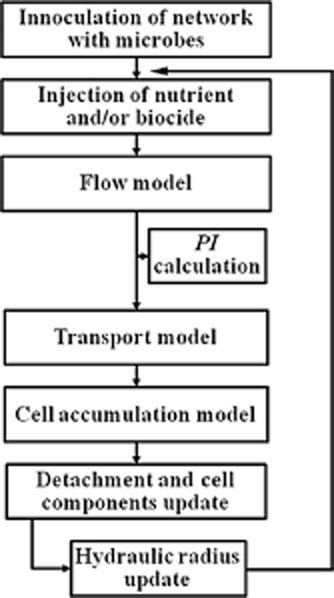
A simplified model algorithm depicting the general sequence of model calculations during each timestep. Biofilm is treated as an adsorbed phase on pore walls and detached biofilms are produced via the network outlet.

The activity of planktonic cells is assumed negligible; therefore, nutrient consumption and growth of planktonic cells are not considered. Concentration fronts are calculated by assuming complete mixing at nodes. Our model uses different diffusion coefficients for inter-pore mass transfer within the water phase (*D_iw_*) and across the water/biofilm (*D_ibf_*) interface. The subscripts *i*, *w* and *bf* represent solute (e.g. biocide, nutrient), water and biofilm respectively. A discretized form of Fick's law is implemented to calculate diffusive transport (Ezeuko *et al*., 2011), whereas the loss of nutrient over time due to biofilm metabolism is determined by Stewart and colleagues (1996):


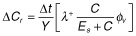
(5)

where *Y* is the yield coefficient (i.e. the ratio of biomass produced per unit of substrate consumed), *λ*^+^ is microbial specific growth rate, *E_s_* is an affinity constant (i.e. the substrate concentration supporting one-half the maximum growth or nutrient uptake rate), and *ϕ_v_* is the non-persister cell concentration (mass per unit pore volume). Nutrient consumption results in biofilm growth and accumulation. To avoid instabilities resulting from discretization of the governing equations, the time step is chosen to be equal to Δ*t* = *V*/*q_max_*; this denotes the global minimum time for flow between nodes in the network. The overall algorithm, presented in Fig. 3, consists of an iterative sequence to simulate biofilm evolution under flow conditions in interconnected porous media.

### Accumulation of bacterial cells and biofilm tolerance to biocides

The availability of bacterial species that can replicate and produce extracellular polymeric substances is crucial to biofilm development. EPS facilitates cell attachment to a solid matrix, as well as the ability of biofilms to maintain a multicellular, structured microbial community (Sutherland, 2001; Flemming and Wingender, 2010). These cell species, which are also generally susceptible to biocide treatment, represent non-persister cells in this work. We model EPS production as a first-order process with respect to cell (i.e. non-persister) growth rate. Based on experimental observations in the literature, (Spoering and Lewis, 2001) we have also considered additional species, which do not consume nutrient or replicate, and do not get killed by biocides; this describes persister cells as used in this work. The growth of non-persister cells is controlled by Monod-type kinetics. Dead cells can accumulate by endogenous decay (first-order dependence on non-persister cell concentration) and by biocide killing of non-persisters. Therefore, without biocide dosing, endogenous decay is the only source of dead cells. We adopt previous models of the rate of biocide action where biocide-based killing is a first-order function of biocide concentration (Stewart *et al*., 1996; Roberts and Stewart, 2005). In practice, biofouling either by planktonic cells or biofilms is typically treated by introducing biocides (i.e. antimicrobial agents). However, microbes in biofilms are significantly more tolerant to biocides compared with their planktonic counterparts (Mah and O'Toole, 2001).

In the absence of biocide, no biocide-induced persisters are formed. Pore-level balances for non-persister cells are controlled by growth, endogenous decay, persister formation, detachment and biocide-based killing rates:


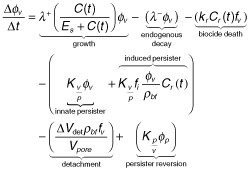
(6)

where *f_v_* is the pore-level volume fractions of non-persister, *K_v/p_* is the specific rate of converting non-persister to persister cells, *K_p/v_* is the specific rate of persister cells reversion to non-persister cells and *f_i_* is a fraction of *K_v/p_* that is induced by biocide. *λ*^+^ is specific growth rate and *λ^−^* is endogenous decay rate, *E_s_* is half saturation constant, *k_r_* is biocide reaction rate, *ϕ_v_* is the concentration of non-persister cells, and *ϕ_p_* is the concentration of persister cells (mass per unit pore volume), *V_pore_* is pore volume, *V_det_* is the volume of shear-detached biofilm, *C_r_*(*t*) is the instantaneous local concentration of biocide, and *ρ_bf_* is the dry biofilm density.

Similarly, the time rate of change of dead cells concentration, *ϕ_d_*, is controlled by the rates of endogenous decay, biocide-based death, and detachment:


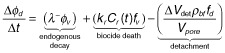
(7)

where *f_d_* is the pore-level volume fraction of dead cells in the biofilm. The rate of accumulation of persisters is given by:


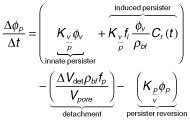
(8)

where *f_p_* is the pore-level volume fraction of persister cells in the biofilm. We adopt our previous model (Ezeuko *et al*., 2011) for biofilm spreading away from a host pore. This model assumes that when a host pore is completely saturated with biofilm, the biofilm can spread into a perimeter pore with the largest spreading potential which is a function of both the local nutrient concentration and local volumetric flow rate. As a result, the leading percolation pathway (or interface) of an expanding biofilm is predicted by this model.

### Critical shear stress and biocide-induced detachment

In this work, biofilm detachment depends on a critical shear stress. Biofilm detachment, therefore, occurs when a predefined biofilm critical shear stress, *τ_crit,_* is exceeded by the pore-level shear stress, *τ*, calculated for cylindrical pores as (Kim and Fogler, 2000):


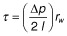
(9)

where *Δp* *l^−1^* is the pressure gradient in a pore of length *l* and *r_w_* is the pore radius open to bulk water flow. The value of the critical shear stress depends on the characteristics of the microbes and the local environment. It is therefore difficult to predict critical shear stress *a priori*. For example, previous investigations have shown that exposure to oxidizing agents can compromise biofilm matrix strength (i.e. reduce biofilm critical shear stress) resulting in enhanced biofilm removal by detachment (DeQueiroz and Day, 2007; Shakeri *et al*., 2007). We assume that biocide completely dissolves in the aqueous phase and represent its impact on critical shear stress as:


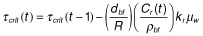
(10)

where *d_bf_* is the biofilm thickness within a pore, *t* is time, *R* is the pore radius, *k_r_* is the biocide reaction rate, and *μ_w_* is the bulk water viscosity. At pore level, when the shear stress exceeds the critical shear stress, the volume rate of biofilm detachment is given by (Kim and Fogler, 2000):


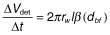
(11)

where *β* is the biofilm detachment coefficient (*t*^*−*1^) – (i.e. biofilm detachment does not occur unless a sufficient biofilm thickness is available to satisfy the volume rate of detachment prescribed by the detachment coefficient). After detachment occurs, the biofilm thickness and bulk water radius, *r_w_*, in the pore are updated by:


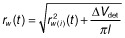
(12)

where


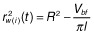
(13)

Equation 12 shows that under suitable conditions, biofilm thickness attained during growth can be eroded by shear detachment. We constrain the equations to ensure that the maximum volume of biofilm that can be detached equals the available biofilm volume within the pore (i.e. a pore matrix does not expand). The composition of each biofilm component (EPS, dead, non-persister and persister cells) in the detached volume is calculated based on the volume fraction of the components in the biofilm (i.e. the most dominant component within the biofilm is also dominant in the detached volume). We assume that detached portions of biofilm do not reattach.

### Simulation model

Table 1 lists the model input parameters used in this study. A 2D network of dimensions 100 by 100 nodes is used for all simulations. Each pore element is assigned a radius, *R*, drawn from a Rayleigh probability distribution function (*R_min_* = 5.5 μm and *R_max_* = 30 μm), representative of a consolidated sandstone reservoir (Wardlaw *et al*., 1987). The concentration of injected nutrient and biocide both equals 1 × 10^−2^ kg m^−3^. The overall pressure gradient across the network for all cases is fixed at 1.6 kPa m^−1^. Table 2 lists the cases evaluated – two categories are examined; a non-layered network (N), and a layered network (L).

**Table 1:** Simulation parameters common to all models

Parameter	Notation	Values	Reference
Density of water	*ρ_w_*	1000 kg m^−3^	Assumed
Density of biofilm (dry)	*ρ_bf_*	20 kg m^−3^	Ro and Neethling (1991)
Viscosity of bulk water	*μ_w_*	10^−3^ Pa s	Assumed
Diffusion coefficients	*D_sw_*	2.7 × 10^−9^ m^2^s^−1^	Roberts and Stewart (2005)
	*D_sbf_*	0.75 *D_sw_*	Assumed
	*D_bw_*	5.0 × 10^−10^ m^2^s^−1^	Roberts and Stewart (2005)
	*D_bbf_*	0.75 *D_bw_*	Assumed
Yield coefficient	*Y*	0.8	Roberts and Stewart (2005)
Maximum specific growth rate	*λ^+^*	1.6 × 10^−4^ s^−1^	Klapper *et al*. (2002)
Maximum specific decay rate	*λ^−^*	1.6 × 10^−6^ s^−1^	*λ^−^* = 0.01 *λ*^+^
Half saturation constant	E*_s_*	1.0 × 10^−4^ kg m^−3^	Roberts and Stewart (2005)
Substrate injection concentration	*C*(0)	1.0 × 10^−2^ kg m^−3^	Assumed
Biocide injection concentration	*C_r_*(0)	1.0 × 10^−2^ kg m^−3^	Roberts and Stewart (2005)
Coefficient of persisters formation (*K_v/p_*) and persister reversal (*K_p/v_*)	*K_v/p_* *=* *K_p/v_*	5.0 × 10^−9^ s^−1^	Assumed
Fixed global pressure gradient	*ΔP_network_*	1.6 kPa m^−1^	Assumed
Biofilm/bulk water viscosity ratio	*α*	10^5^	See Eq. 1

**Table 2:** List of pore network model cases (dimensions are 100 × 100 nodes; NX denotes non-layered models whereas LX denotes layered networks)

Case name	Biocide	Biocide dosing start (h)	Detachment?	*β* (s^−1^)	*K_r_* (s^−1^)	Note
N1	No	–	No	–	Nil	
N2	No	–	Yes	1.0 × 10^−5^	Nil	
N3	No	–	Yes	1.0 × 10^−3^	Nil	
N4	Yes	2.5	Yes	1.0 × 10^−5^	1.0 × 10^−3^	Single cycle biocide dosing for 12 h
N5	Yes	5	Yes	1.0 × 10^−5^	1.0 × 10^−3^	Two cycles of biocide dosing, each lasting 12 h (5 h interval between dosing cycles)
L1	Yes	2.5	Yes	1.0 × 10^−5^	1.0 × 10^−3^	Single cycle biocide dosing for 12 h
L2	Yes	144	Yes	1.0 × 10^−5^	1.0 × 10^−3^	Single cycle biocide dosing for 12 h

The non-layered network is used to investigate effects of shear stress detachment and biocide dosing. Non-persister cells (pore-level cell concentration of 1.0 × 10^−8^ kg m^−3^) are seeded at 60 randomly selected sites (away from the inlet) at the start of the simulation, to mimic bacterial cell attachment. This allows us to focus our discussions on biofilm evolution without additional complications of planktonic cell transport. In Cases N1, N2 and N3, the effect of shear detachment is evaluated by comparing simulations with and without detachment in the absence of biocide dosing. Case N4 and N5 are used to investigate the impact of biocide; in case, N4, 2.5 h of nutrient injection is followed by 12 h of biocide dosing, while in case N5, 5 h of nutrient injection is followed by two 12 h biocide dosing cycles. Nutrient injection is discontinued during biocide dosing.

**Fig. 4:**
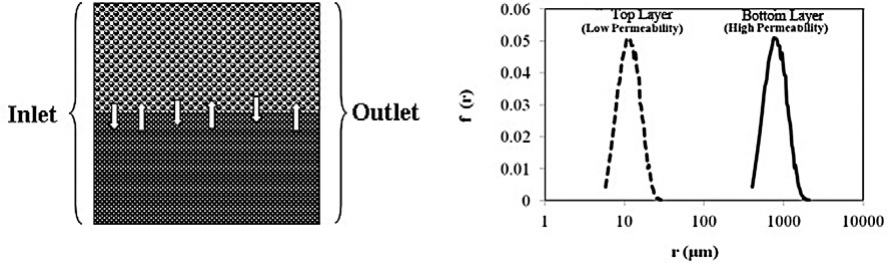
Schematic of a layered network used to idealize artificially induced or geologically intrinsic layering in subsurface porous systems. The white arrows indicate that a communication (liquid flow, biofilm growth) exists between the two layers. They do not necessarily suggest the direction of liquid or biofilm movement. Pore-size distribution of the top layer is representative of a consolidated sandstone reservoir rock.

To mimic geologically intrinsic or artificially induced (e.g. hydraulic fracture filled with beads) permeability layering (Cases L1 and L2), we design a network model with two sections in which pore radii in one of the sections are increased by a factor of seventy compared with the other section, while retaining the original pore size distribution (see Fig. 4). This simplified model contains both pore and layer-scale heterogeneity. Similar to the non-layered cases, we seed non-persister cells at 60 randomly selected sites in the network, away from the inlet.

Productivity index, *PI*, is a typical quantitative parameter used in the petroleum industry to evaluate the subsurface flow efficacy of hydrocarbons through a reservoir cross-section into a production wellbore. For a given pressure drawdown, the productivity index is calculated by (Dake, 2001):


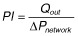
(14)

where *Q_out_* is the outlet flow rate and *ΔP_network_* is the fixed pressure drop across the network. In this paper, we adopt the concept of productivity index for quantifying formation damage associated with biofilm growth.

## Results and discussion

We adopt a bacterial growth rate consistent with a measured biofilm bacteria doubling time of 1.2 h as reported in Klapper and colleagues (2002). It is also important to clarify that the simulations and results presented here are intended to provide a pore-level (millimetre to centimetre scale) insight into phenomena that are typically discussed at much larger scales (i.e. metres). As a result, appropriate upscaling is still essential for any form of quantitative comparison with field scale. However, the understanding afforded by pore-level modelling reveals important trends that must be captured by selected upscaling methods.

### Cases N1, N2 and N3: shear stress biofilm detachment

**Fig. 5:**
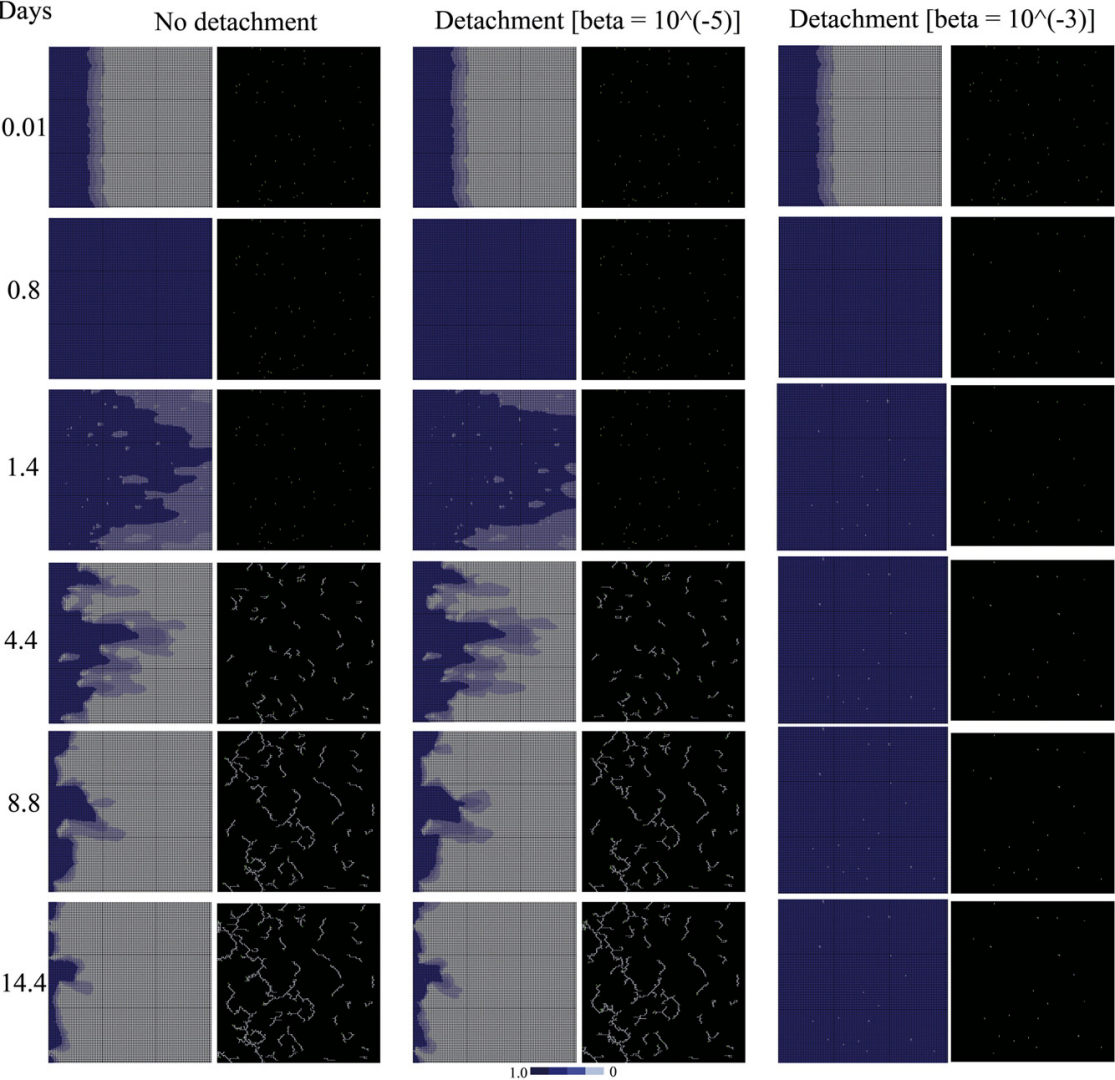
Simulated time evolution of spatial nutrient concentration and biofilm morphology for Cases N1, N2 and N3 with and without biofilm detachment in a 2.5 cm × 2.5 cm model. Nutrient concentration is dimensionless with respect to injected nutrient concentration. Principal flow direction is from left (inlet) to right (outlet). For each case, the first column shows nutrient concentration in the network whereas the second column shows biofilm morphology – aqueous phase pores are shown as black while biofilm-saturated pores are shown as green/white [seeded biofilm sites = 60; no biocide].

It is important to clarify that the network seed (i.e. the spatial distribution of pore geometry and local connectivity) and the initial cell attachment sites in all non-layered cases are exactly the same. Any difference in results can therefore not be attributed to seeding effects. Simulations were run for about 15 days, i.e., typical duration of experimental studies. Figure 5 displays nutrient concentration and biofilm spatial distributions, and compares models with and without biofilm detachment. The small concentration of attached cells at the start of each simulation results in a low rate of nutrient consumption in comparison with the rate of nutrient influx. Consequently, the nutrient concentration reaches maximum values throughout the network at early stages (about 0.9 h) of the evolution in the pore network. A concentration gradient eventually emerges as the biofilms grow larger and consume a large fraction of the available nutrient. In addition, bioclogging of pores impedes nutrient supply, resulting in nutrient-limited growth downstream of the network. This biases biofilm growth towards the nutrient source (which is the inlet). The inclusion of detachment results in a less dense biofilm. For the smaller detachment rate case (β = 1.0 × 10^−5^ s^−1^), the impact of biofilm detachment on biofilm development is more concentrated around the inlet where flow velocity is larger (fixed pressure drop across network). Results however show that very large detachment rate (representative of very weak biofilms) leads to very poor accumulation of biofilm in porous media and in certain cases, complete removal of biofilms from seeded sites – Case N3 (β = 1.0 × 10^−3^ s^−1^). The implication of detachment for biofilm control is therefore obvious from these results.

**Fig. 6:**
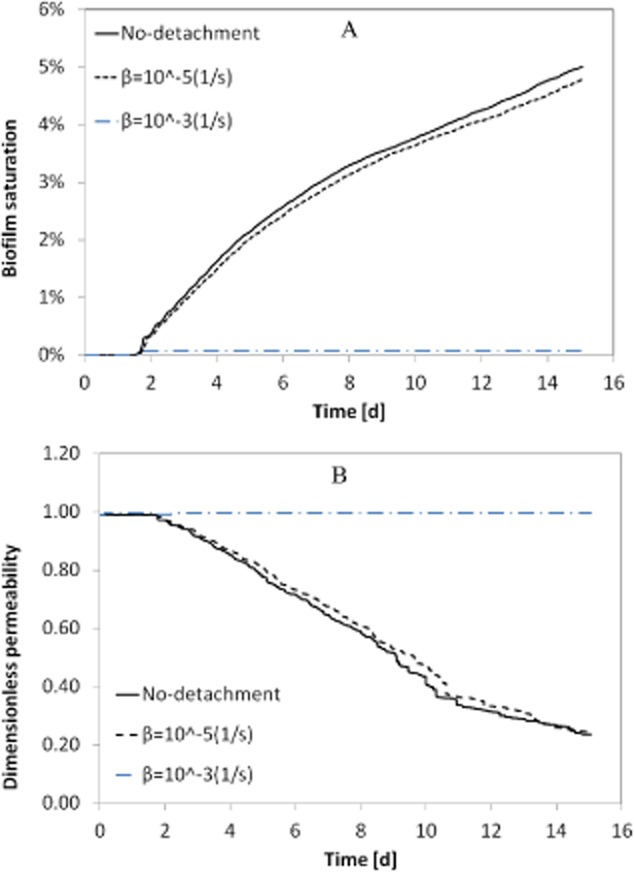
Time evolution of network biofilm saturation for cases N1, N2 and N3. The solid curve indicates simulation without biofilm detachment (Case N1), whereas the dashed line represents simulation with biofilm detachment (Cases N2 and N3) – [seeded biofilm sites = 60; no biocide]. A. Network biofilm saturation versus time. B. Dimensionless permeability versus time.

In Fig. 6, quantitative model results of biofilm saturation and dimensionless permeability is presented and shows that little or no detachment occurs during the early growth period when biofilm thickness has not yet reached the threshold for detachment (see Eq. 11). The network biofilm saturation in Fig. 6 shows that for the presented biofilm detachment rates, biofilm growth in the absence of detachment surpasses that with detachment. Figure 6 shows that very large biofilm detachment rate results in a consistent minimal damage to the permeability of the porous media (Case N3, β = 1.0 × 10^−3^ s^−1^). Under a fixed external pressure gradient, local shear forces drop (reducing the possibility of detachment) with greater accumulation of biofilm. This reduction in detachment results in eventual convergence of the dimensionless permeability for cases without detachment (case N1) and that with relatively small detachment Case N2. These rather intuitive results consolidate our confidence in the model.

### Cases N4–N5: biocide dosing and biofilm regrowth

**Fig. 7:**
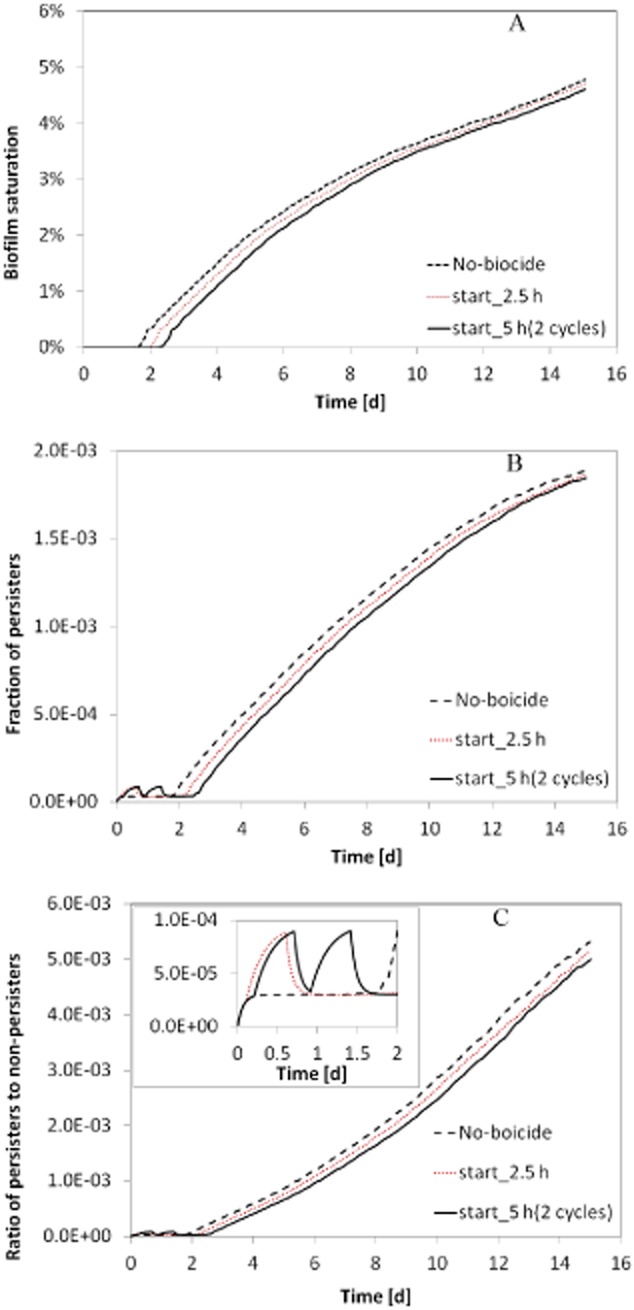
Temporal evolution of (A) network biofilm saturation in Cases N2, N4 and N5; (B) fraction of persisters in the network and (C) ratio of persisters to non-persisters in the network. Inset shows the ratio of persisters to non-persisters over the biocide-treatment period. [*K*_*v/p*_ = *K*_*p/v*_ = 5.0 × 10^−9^ s^−1^; *K*_*r*_ = 1.0 × 10^−3^ s^−1^; seeded biofilm sites = 60; *β* = 1.0 × 10^−5^ s^−1^].

For applications where the ultimate aim is to control the growth of biofilms in porous media, it appears intuitive that biocide treatment of biofilms should be implemented at early stages when biofilm accumulation is very low. Two cases (N4 and N5) are used to evaluate the efficacy of biocide when treatment is effected after a few hours of fluid injection. In Case N4, 12 h of biocide dosing starts after 2.5 h of nutrient injection. Case N5 however evaluates early biocide dosing commencing after 5 h of nutrient injection and involving two treatment cycles, separated by 5 h interval. Case N2 models a scenario without biocide dosing. In both cases N4 and N5, buffer solution without dissolved nutrient are injected during biocide treatment, and small biofilm detachment rate equals to that for case N2 (β = 1.0 × 10^−5^) are adopted. Figure 7 shows that there is a suppression of biofilm accumulation resultant from biocide treatment. This early suppression of biofilm accumulation rate is associated with non-persister cells being killed by biocide. Specifically, the concentration of dead cells in biofilm increases and eventually becomes the dominant fraction. However, the biofilm rate of accumulation eventually rises (attaining slopes similar to the untreated case) when biocide dosing terminates. Conversely, without biocide dosing (case N2), only endogenous cell decay occurs and the fraction of persisters increases with time (Fig. 7B). An increase in persister cells fraction is indicative of an increase in biofilm tolerance to biocide. Therefore, the simulated untreated case also indicates a decrease in biofilm susceptibility to biocide treatment with an increase in time (i.e. aging of biofilm). This is in agreement with previous experimental observations which show that susceptibility of biofilms to biocide decreases with aging of biofilms (Anwar *et al*., 1992; Foley and Gilbert, 1997). For the untreated case, or in parts of the network yet to be contacted by biocide, the increase in the persister/non-persister fraction over time is related to the formation of persisters and the effective reduction in non-persisters due to endogenous decay. Whereas persisters tolerate biocide treatment, they do not proliferate in the presence of biocide even if nutrient is present. Therefore, for the biocide treated system, the increase in the persister/non-persister fraction – in areas of the network that are contacted by biocide – is a result of an effective decline in the concentration of non-persisters due to both biocide exposure and endogenous decay. Figure 7 shows that over time, the persister/non-persister ratio for an untreated system can surpass that of a biocide treated system. The inset in Fig. 7 highlights the persister/non-persister fraction over time covering the period of biocide treatments and shows a drastic increase in this ratio during biocide treatment. Although biocide treatment was implemented very early, its overall effect of controlling biofilm accumulation is significantly poorer than those typically reported for bulk experiments.

For a homogeneous medium where permeability reduction is undesirable in any region (such as regions that have been hydraulically fractured and packed with beads), this prediction corroborates earlier experimental observations (Shaw *et al*., 1985) showing that only killing of biofilm bacteria by biocide has a small effect on checking biofouling of porous media. Effective biofilm control requires biocides such as strong oxidizing agents that ultimately facilitate increased detachment of biofilms. As discussed in the next paragraph, this model offers a basis for evaluating qualitative behaviours under a number of varying parameters including the interaction of biofilm growth, flow diversion, spatial biofilm topology and productivity index in layered media. Further studies that address the evaluation of biocide dosing protocols (e.g. repetitive, continuous) are currently underway in our laboratory.

### Cases L1 and L2: biofilm growth in a layered network

**Fig. 8:**
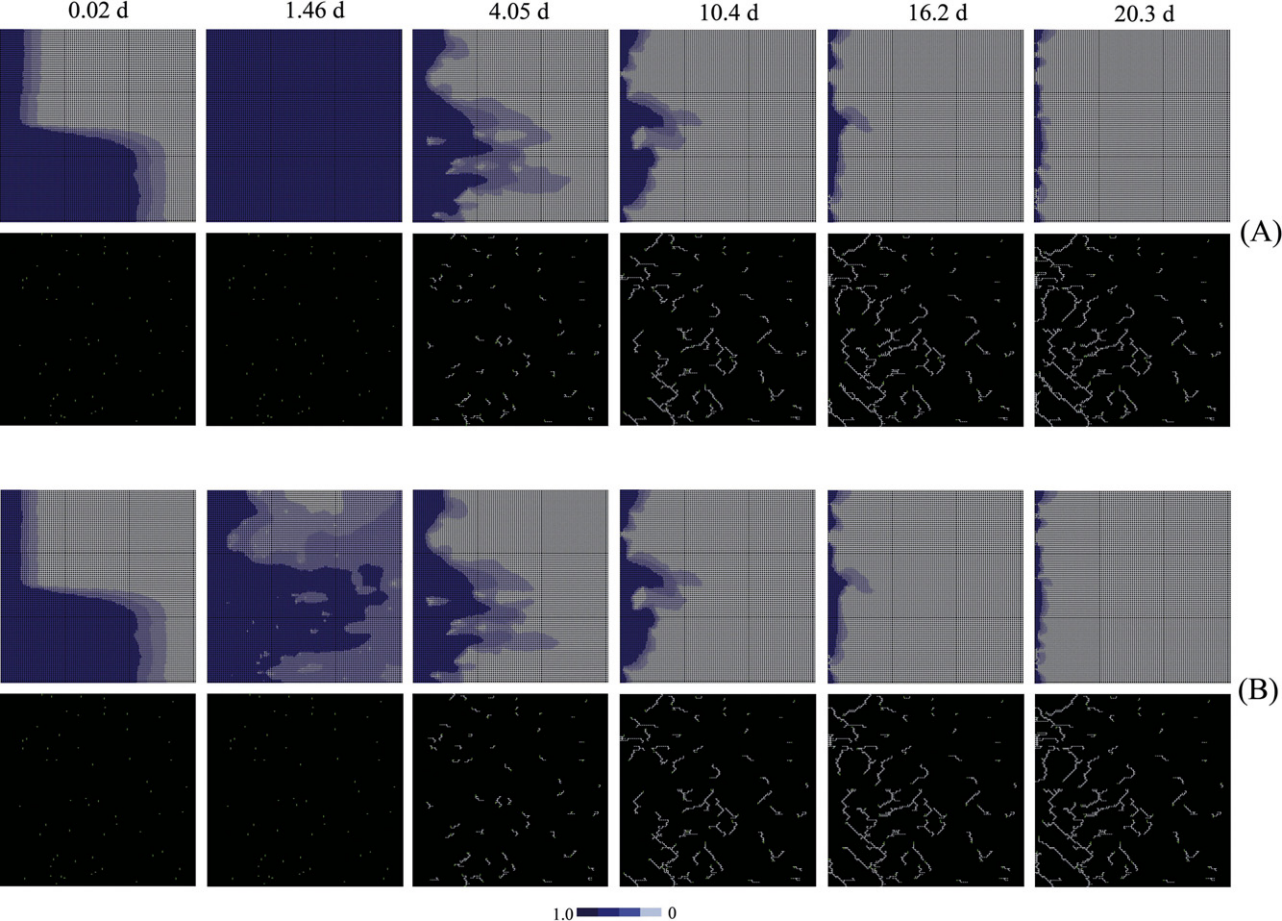
Time evolution of spatial nutrient concentration (upper images) and biofilm morphology (lower images) for Cases L1 and L2 layered network in 2.5 × 2.5 cm model. In each network, pore sizes of the bottom layer have been multiplied by 70. Nutrient concentration is dimensionless with respect to injected nutrient concentration. The principal flow direction is from left (inlet) to right (outlet). Aqueous phase pores are shown in black whereas biofilm-saturated pores are shown in white [seeded biofilm sites = 60, in top layer only; *β* = 1.0 × 10^−5^ s^−1^]. A. Case L1. B. Case L2.

As shown in Fig. 8, the nutrient front in a layered network exhibits a dominant finger after 0.19 h of simulated time. However, the whole network eventually becomes saturated with nutrient. Figure 8 shows nutrient concentration and biofilm occupancies for case L1 (i.e. biocide injection commencing after 2.5 h) while Fig. 8 shows results for case L2 (i.e. biocide injection commencing after 6 days). In cases L1 and L2, biofilm is seeded in both the top and bottom layers. The results show that the biofilm enlarges and clogs the upstream region of both layers, causing nutrient-limited growth in the downstream region of each layer. The effect is preferential growth towards the nutrient supply which increases bioclogging towards the inlet of each layer. Biofilm accumulation however occurs at a faster rate within the higher permeability bottom layer. Results here indicate that for conformance problems where the aim of the process is to reduce flow from the high permeability bottom layer (as may be desirable during waterflooding in oil fields or bioremediation by bioclogging to minimize the transport rate of underground contaminants), then permeability damage of the bottom layer will be considered an improvement in process productivity (i.e. water is channelled to the lower permeability layer). However, permeability damage of the top layer for such a case will represents a decline in productivity and can be described as formation damage.

**Fig. 9:**
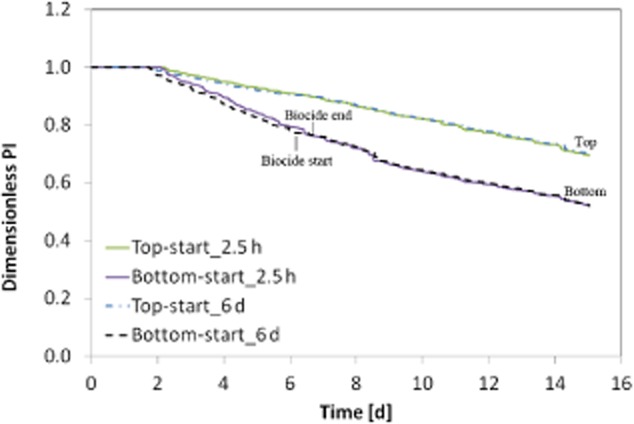
Time evolution of dimensionless *PI* [*PI(t)/PI(0)*] in top (low permeability) and bottom (high permeability) layers. Pore sizes of the bottom layer have been multiplied by 70 [seeded biofilm sites = 60; *β* = 1.0 × 10^−5^ s^−1^].

A plot of time-lapse dimensionless *PI* for each layer, shown in Fig. 9, shows that while greater permeability damage occurs in the bottom layer, the permeability of the top layer is also damaged.

It is important to clarify that, while layer permeability also offers an alternative quantifier of the performance of such processes, we discuss productivity index because it is easier to evaluate – permeability calculations from permanent downhole gauges (e.g. well analysis) is currently very challenging. Results from our layered network therefore suggest that biological permeability conformance using biofilms may lead to formation damage by clogging the undesired layer within the porous media. These results also show that late-time initiation of biocide treatment offers negligible improvement for bioclogged porous medium.

## Conclusions

The numerical model presented in this study is, to our knowledge, the first to have incorporated the tolerance of biofilms to biocide based on the presence of persister cells, coupled with flow, transport and reaction in interconnected porous media. Furthermore, the generation of persister cells, both through innate as well as biocide-induced processes, were included based on emerging knowledge of biocide action on biofilms.

To evaluate biofilm-induced formation damage related to processes such as hydraulic fracturing, waterflood conformance treatment and bioremediation, we considered models with pore-scale and layer-scale heterogeneity. Based on 2D simulation results, we conclude that treatment with biocide can offer a temporary respite to bioclogging due to the presence of persister cells. By adopting the persister cells concept, the predicted increase in the persister cell fraction with simulation time is consistent with previous experimental reports of increased biofilm tolerance with aging. Under the fixed and relatively small pressure gradient conditions used, a permeability increase did not occur during biocide treatment. Therefore, effective treatment of biofilm-induced formation damage in porous media perhaps requires not only the killing of bacterial cells, but also the removal of the EPS matrix. This may be achieved by the introduction of a sufficiently large external shear stress to the biofilms or the application of treatments that weaken biofilms making them easily detachable at low pressure gradients.

Finally, successful application of microbial conformance treatment is more complicated than simply engineering the attachment of biofilm-forming cells – appropriate design of biocide and nutrient supply protocols must therefore be sought. The implications of these results motivate further experiments of bioengineered permeability conformance in systems with inter-layer flow communication. Experimental data will also improve model predictions of biofilm-induced formation damage. Our future research focus includes an in-depth investigation into finding the best operational protocols for effective control and minimization of formation damage associated with engineered biofilm growth in layered systems.

## Nomenclature

C = nutrient concentration (kg m^−3^)

*C*_*r*_ = biocide concentration (kg m^−3^)

*d*_*bf*_ = pore-level biofilm thickness (m)

*D*_*bbf*_ = Diffusion coefficient of biocide across biofilm (m^2^ s^−1^)

*D*_*bw*_ = Diffusion coefficient of biocide across water (m^2^ s^−1^)

*D*_*sbf*_ = Diffusion coefficient of nutrient across biofilm (m^2^ s^−1^)

*D*_*sw*_ = Diffusion coefficient of nutrient across water (m^2^ s^−1^)

*E*_*eps*_ = EPS mass per unit pore volume (kg m^−3^)

*E*_*s*_ = half saturation constant (dimensionless)

*f*_*d*_ = pore-level volume fraction of dead cell in biofilm (dimensionless)

*f*_*eps*_ = pore-level volume fraction of EPS in biofilm (dimensionless)

*f*_*i*_ = fraction of *K*_*v/p*_ that is induced by biocide (dimensionless)

*f*_*p*_ = pore-level volume fraction of persister in biofilm (dimensionless)

*f*_*v*_ = pore-level volume fraction of non-persister cells in biofilm (dimensionless)

K = Network permeability (m^2^)

*K*_*eps*_ = coefficient of EPS production (s^−1^)

*K*_*p/v*_ = coefficient of reversion; persisters to non-persister cells (s^−1^)

*k*_*r*_ = biocide reaction rate (s^−1^)

*K*_*v/p*_ = coefficient of conversion; non-persister cells to persisters (s^−1^)

*l* = pore length (m)

*Δp* = inter – pore pressure gradient (Pa)

*ΔP*_*network*_ = global pressure gradient (Pa)

*q* = pore-level flow rate (m^3^ s^−1^)

*Q*_*out*_ = outlet flow rate (m^3^ s^−1^)

*R* = radius of pore with zero biofilm (m)

*r*_*w*_ = pore-level radius open to bulk water flow (m)

*Δt* = simulation time step (s)

*V*_*bf*_ = pore-level volume of biofilm (m^−3^)

*V*_*det*_ = volume of detached biofilm within a pore (m^−3^)

*V*_*pore*_ = pore volume (m^−3^)

*Y* = yield coefficient (dimensionless)

## Greek letters

*μ*_*bf*_ = biofilm water viscosity (Pa.s)

*μ*_*w*_ = bulk water viscosity (Pa.s)

*ϕ*_*d*_ = dead cell mass per unit pore volume (kg m^−3^)

*ϕ*_*p*_ = persister cell mass per unit pore volume (kg m^−3^)

*ϕ*_*v*_ = non-persister cell mass per unit pore volume (kg m^−3^)

*α* = ratio of biofilm water to bulk water viscosity (dimensionless)

*β* = biofilm detachment coefficient (s^−1^)

*λ*^*−*^ = endogenous decay rate (s^−1^)

*λ*^*+*^ = maximum specific growth rate (s^−1^)

*ρ*_*bf*_ = biofilm dry density (kg m^−3^)

*τ* = pore-level shear stress (Pa)

*τ*_*crit*_ = critical shear stress (Pa)

## Abbreviations

EPS: extracellular polymeric substance
PI: productivity index
PNM: pore network model
